# SynBioGPT2: A dynamic reasoning framework enables high-fidelity design of microbial cell factories

**DOI:** 10.1016/j.bidere.2026.100093

**Published:** 2026-06-25

**Authors:** Zhitao Mao, Jun Du, Jirun Guan, Wei Wang, Ruoyu Wang, Haoran Li, Zhenkun Shi, Qianqian Yuan, Xiaoping Liao, Hongwu Ma

**Affiliations:** a Biodesign Center, Key Laboratory of Engineering Biology for Low-carbon Manufacturing, Tianjin Institute of Industrial Biotechnology, Chinese Academy of Sciences, Tianjin, 300308, China; b Haihe Laboratory of Synthetic Biology, Tianjin, 300308, China; c National Technology Innovation Center of Synthetic Biology, Tianjin, 300308, China; d State Key Laboratory of Medicinal Chemical Biology, Frontiers Science Center for Cell Responses, College of Life Sciences, Nankai University, Tianjin, 300071, China

**Keywords:** Large language models (LLMs), Retrieval-augmented generation (RAG), Dynamic reasoning, Prompt engineering, Rational strain design

## Abstract

The rational design of microbial cell factories is essential for sustainable biomanufacturing, yet the traditional Design-Build-Test-Learn (DBTL) cycle is bottlenecked by the highly non-linear and interconnected nature of biological systems. Although large language models (LLMs) offer computational advantages for automated design, their application in systems metabolic engineering is hindered by factual inconsistencies and a limited capacity for multi-hop causal reasoning—challenges that static single-pass retrieval-augmented generation (RAG) fails to resolve. Here, we present SynBioGPT2, a dynamic reasoning framework that integrates paragraph-level hybrid retrieval, an iterative self-evaluation loop, and domain-specific expert prompt templates to enable autonomous, multi-source knowledge synthesis. Evaluated on a multidimensional synthetic biology benchmark, the architecture achieved 91.67% accuracy and completeness, significantly outperforming zero-shot LLMs and static RAG baselines. We demonstrated the framework's capability to resolve systems-level biochemical constraints, including redox balancing and complex allosteric feedback networks, during the rational computational design of *Corynebacterium glutamicum*. Furthermore, SynBioGPT2 ensured the high-fidelity extraction of quantitative parameters and successfully reconstructed 93.5% (86/92) of expert-curated metabolic engineering strategies across diverse target products. By mitigating structural reasoning deficits and integrating expert-guided deductive logic, SynBioGPT2 provides a mechanistically robust, scalable platform to accelerate automated biological discovery and rational cell factory engineering.

## 1 Introduction

The rational design of microbial cell factories is central to advancing sustainable biomanufacturing [1,2]. However, bridging the gap between conceptual metabolic design and scalable industrial phenotypes remains a formidable challenge [[3], [4], [5], [6]]. Current strain engineering predominantly relies on the empirical Design-Build-Test-Learn (DBTL) cycle [7,8]. Despite advancements in high-throughput multi-omics and precise genome editing, this iterative process is severely constrained by the highly interconnected and non-linear nature of cellular metabolism [9]. Genetic perturbations frequently induce unpredictable systemic responses—such as flux redistribution, redox imbalances, and the accumulation of toxic intermediates—rendering traditional trial-and-error approaches insufficient for modern efficiency requirements [10]. Consequently, there is an urgent need for advanced computational frameworks capable of rationally navigating this vast, multidimensional perturbation space.

Large language models (LLMs) have recently emerged as powerful computational tools for scientific knowledge extraction and hypothesis generation [[11], [12], [13]]. While these models excel at natural language processing, their direct application to systems metabolic engineering is fundamentally limited [14,15]. Generalized LLMs rely on static parametric memory, rendering them incapable of integrating rapidly evolving frontier research. More critically, when tasked with complex, multi-hop biological reasoning—which requires integrating enzyme kinetics, regulatory networks, and host-specific compatibility—these models frequently generate factual inconsistencies. In the context of metabolic engineering, a single misidentified gene locus, an overlooked allosteric regulator, and/or a species mismatch can invalidate an entire pathway design, strictly limiting the utility of standalone LLMs in rigorous scientific workflows.

To address these reliability deficits, retrieval-augmented generation (RAG) has been adopted to ground model outputs in external literature, thereby mitigating hallucination risks [[16], [17], [18], [19]]. However, even advanced static RAG architectures—including those deploying two-stage hybrid retrieval—operate on a single-pass paradigm. While these advanced systems successfully capture both lexical and semantic relevance, they remain structurally static. Consequently, in high-precision synthetic biology tasks, these single-pass pipelines frequently fail to dynamically assemble fragmented logical chains across multiple documents, leading to pathway-context misalignment and information contamination.

While these static architectures have been widely adopted, recent advances have significantly expanded the AI toolkit available to synthetic biologists. Critically, however, the majority of these innovations remain structurally constrained by single-pass paradigms, unable to dynamically refine outputs through iterative self-evaluation. At the protein-engineering level, specialized protein language models (pLMs) such as ESM-3 [20] and ProGen2 [21] now enable zero-shot structure prediction and fitness landscape navigation, complementing sequence-based metabolic design with structural insights; yet these models operate without iterative factual verification against external literature or metabolic network context. At the knowledge-retrieval level, domain-specific RAG systems—including NEKO (Network for Knowledge Organization), which integrates knowledge graphs with LLMs for synthetic biology research [22], and RAGulate, which provides post-hoc regulatory assessment grounded in automated literature retrieval [17]—have improved corpus coverage and semantic precision; nevertheless, they retrieve evidence once without dynamically regenerating sub-queries based on intermediate biochemical findings. At the general-reasoning level, advanced models such as DeepSeek-R1 [23] and Claude-Sonnet-4 have demonstrated substantially enhanced chain-of-thought capabilities for scientific hypothesis generation; however, they lack domain-specific, structured self-evaluation loops to resolve multi-hop biochemical dependencies across fragmented literature. Collectively, these parallel advances underscore a persistent, unmet need: the transition from static, single-pass information processing to dynamic, iterative reasoning frameworks capable of autonomously assembling and validating complex metabolic engineering strategies across multiple knowledge sources.

Here, we present SynBioGPT2 (https://synbiogpt.biodesign.ac.cn/), a dynamic computational architecture engineered to overcome the structural reasoning deficits of existing AI models in metabolic engineering. Moving beyond static retrieval pipelines, SynBioGPT2 integrates paragraph-level hybrid indexing with a dynamic reasoning loop—enabling iterative query decomposition, self-evaluation, and rigorous factual constraints. By integrating rigorous biological constraints into the inference loop, this framework systematically mitigates factual inconsistencies and reconstructs complex metabolic engineering strategies with high-fidelity. Here, 'high-fidelity' refers to the framework's capacity to generate quantitatively precise and mechanistically accurate engineering strategies that faithfully reflect established biochemical constraints, rather than implying a perfect replication of a pre-existing biological template. This study provides a robust methodological paradigm for human-AI collaborative strain engineering, establishing a definitive computational foundation for deeper AI-driven applications in target discovery, pathway optimization, and automated biological design.

## 2 Methods

### 2.1 System architecture

SynBioGPT2 is implemented as a three-layer modular architecture consisting of a Data Layer, an Index Layer, and an Inference Layer. The Data Layer is responsible for literature acquisition, parsing, and structured preprocessing. The Index Layer constructs a paragraph-level hybrid retrieval system. The Inference Layer performs dynamic query decomposition, iterative reasoning, and answer validation. These components operate independently but are integrated into an end-to-end pipeline for knowledge retrieval and generation in synthetic biology applications.

To contextualize the architectural upgrades in SynBioGPT2, we briefly contrast it with its predecessor, SynBioGPT1. SynBioGPT1 employed a conventional dense-vector RAG framework that, while capturing broad semantic associations, frequently retrieved contextually irrelevant passages for precision-critical metabolic engineering queries [19]. SynBioGPT2 addresses these limitations through three structural upgrades (see Supplementary Table 1 for a detailed comparison): (i) paragraph-level hybrid indexing replaces document-level dense retrieval with a BM25 + fine-tuned BERT ensemble, improving precision for domain-specific entities; (ii) an iterative self-evaluation loop replaces the single-pass paradigm, enabling dynamic query refinement based on intermediate evidence quality; and (iii) an enhanced Data Layer with advanced OCR and table reconstruction ensures higher-fidelity quantitative extraction. These modifications collectively advance mechanistic accuracy and domain alignment beyond the capabilities of SynBioGPT1.

### 2.2 Data collection and preprocessing

A domain-specific corpus was constructed from open-access publications in synthetic biology and systems metabolic engineering. Articles and associated metadata were collected from major repositories, including Web of Science and PubMed Central, using compliant automated scripts and API-based retrieval. The assembled corpus comprises 69,593 full-text articles and 27,430 abstracts as of June 2026. To ensure the knowledge base remains current, we implemented a monthly automated update protocol: newly published articles matching predefined keyword filters are retrieved via API queries and processed through the established parsing pipeline described below, with outputs incrementally indexed into the vector database.

Raw PDF documents were processed using a Docling-based pipeline. To address common parsing challenges in life science literature—such as complex tables, multi-page layouts, and dense reference sections—the pipeline integrates optical character recognition (OCR) and table reconstruction algorithms. This enables accurate extraction of quantitative information, including enzyme kinetic parameters (e.g., Km and *k*_cat_), mutation site annotations, and fermentation performance metrics.

The extracted text was further processed using spaCyLayout (https://github.com/explosion/spacy-layout) for semantic segmentation and structural cleaning. Non-content elements, including headers, footers, copyright notices, acknowledgments, and references, were removed. Structured metadata, such as titles, abstracts, and author affiliations, were retained. The final corpus was converted into a structured Markdown format with associated metadata annotations for downstream indexing and retrieval.

### 2.3 Paragraph-level indexing and hybrid retrieval

To improve retrieval precision, text was indexed at the paragraph-level rather than at the document level. Each paragraph was treated as an independent retrieval unit and stored with its metadata in a PostgreSQL-based vector database.

A hybrid retrieval framework combining sparse and dense indexing was implemented. Sparse retrieval was based on the BM25 algorithm, enabling high-precision matching of domain-specific entities such as gene names, mutation identifiers, and chemical compounds. Dense retrieval employed a BERT-based encoder adapted to metabolic engineering corpora to generate contextual embeddings, enabling semantic matching across synonymous expressions and related concepts.

Retrieval results from both methods were combined using Reciprocal Rank Fusion (RRF). This approach improves recall while maintaining precision, and reduces the inclusion of contextually irrelevant passages.

### 2.4 Inference workflow

The inference layer is implemented using a multi-agent framework based on smolagents. For complex queries, a master agent first interprets the overall task and decomposes it into a sequence of sub-queries. These sub-queries correspond to distinct aspects of the problem, such as pathway bottlenecks, cofactor balance, or competing metabolic fluxes.

Each sub-query triggers a retrieval–generation cycle. Relevant evidence is retrieved from the paragraph-level hybrid retrieval system, and intermediate answers are generated by the LLM. A self-evaluation step is then applied to assess factual consistency, logical coherence, and evidence coverage.

The evaluation combines two signals: (i) structured scoring using a LLM acting as a judge (LLM-as-a-Judge), and (ii) token-level log-probabilities from the generation process. If the aggregated confidence score is below a predefined threshold (0.8), the current answer is rejected, and a new sub-query is generated to address the identified knowledge gap.

The system performs up to eight iterative retrieval–reasoning cycles. This constraint limits context accumulation and ensures computational efficiency while allowing sufficient reasoning depth. The final output is generated after integrating evidence across iterations.

### 2.5 Prompt template design

LLMs typically exhibit high sensitivity to input variance, which can compromise the reproducibility and biological validity of generated solutions in specialized domains. To standardize user-system interactions and ensure consistent outputs within the SynBioGPT2 framework, we developed a prompt template consisting of 16 predefined, expert-curated templates (Supplementary File 2).

Rather than expecting users to formulate complex queries from scratch, these templates are systematically organized to mirror the established logical workflow of metabolic engineering strain design. These templates comprehensively cover four sequential dimensions of the developmental lifecycle: Host Selection, Metabolic Pathway Engineering, Metabolic Pathway Regulation, and Experimental Condition Optimization.

By utilizing this structured questioning framework, users are guided to interact with SynBioGPT2 in a highly targeted manner. This method effectively translates the user's biological intent into optimal computational prompts. Consequently, it minimizes the ambiguity of open-ended queries and ensures that the model provides actionable, mechanistically rigorous, and highly specific metabolic modification strategies, thereby significantly enhancing the efficiency and reliability of the human-AI collaborative design process.

### 2.6 Implementation of comparative baselines

To systematically evaluate the architectural contributions of SynBioGPT2, three distinct configurations were implemented for the ablation study. Baseline 1 represents the standalone LLM relying solely on its internal pre-trained parametric knowledge (zero-shot setting). For comparative analysis, a standard document-level RAG architecture (Baseline 2) was implemented. This baseline employed a two-stage hybrid retrieval strategy to ensure both lexical and semantic coverage. In the first stage (coarse-grained recall), a BM25-based keyword search was executed via ParadeDB to retrieve the top 20 candidate document segments. In the second stage (fine-grained re-ranking), the jina-embeddings-v3 model was utilized to calculate vector similarity for the retrieved candidates. The top 5 segments with the highest similarity scores were ultimately selected for the generation phase. All metadata and vector embeddings were managed within a PostgreSQL-based vector database. This configuration represents the typical deployment of LLMs with external knowledge bases prior to the introduction of our dynamic reasoning loop. Finally, Baseline 3 represents the complete SynBioGPT2 architecture, incorporating paragraph-level hybrid retrieval and iterative self-evaluation.

### 2.7 Benchmark construction and evaluation

A benchmark dataset consisting of 120 tasks was constructed to evaluate system performance in microbial strain engineering. Tasks were categorized into four types: (i) factual extraction, requiring precise retrieval of quantitative or structured data; (ii) multi-hop reasoning, requiring integration of evidence across multiple sources; (iii) comprehensive/explanatory tasks, assessing mechanistic understanding; and (iv) counterfactual tasks, designed to test the model's ability to identify and reject incorrect biological assumptions.

To ensure a rigorous, unbiased, and scalable evaluation, we implemented an LLM-as-a-Judge pipeline utilizing Gemini 3.1 Pro as an independent, third-party evaluator (see Supplementary File 3 for the evaluation prompts). By employing an evaluator model architecturally distinct from both generative baselines (DeepSeek-R1 and Claude-Sonnet-4), we explicitly mitigated the self-preference bias frequently observed when models evaluate their own outputs or those of structurally similar variants. Under a blinded evaluation protocol, two binary metrics were defined: accuracy and completeness. A response was considered correct only if it contained no factual errors and included all key biological elements.

To validate the robustness of the LLM-as-a-Judge pipeline, we performed a blind human-expert evaluation using a stratified random sample of 20 tasks (5 tasks randomly selected from each of the four evaluation categories). The human-expert evaluations demonstrated high consistency with the Gemini 3.1 Pro judgments, confirming the reliability of the automated evaluation framework across diverse task complexities.

### 2.8 Quantitative metrics for benchmarking design reconstruction and expansion

To rigorously assess the fidelity and creative capacity of SynBioGPT2, we employed two distinct metrics to quantify its performance against the expert-curated benchmark. The first metric, Overlap Recovery Rate, was defined to evaluate the model's ability to accurately reconstruct validated, expert-level biochemical interventions. This rate is calculated as the percentage of literature-reported strategies ($N_{\text{literature}}$) that were successfully reproduced by the model ($N_{\text{overlap}}$), specifically: Recovery Rate (%) = $\left(N_{\text{overlap}}/N_{\text{literature}}\right)\times 100\%$. A high recovery rate serves as a primary indicator of the system's knowledge-driven reliability and its ability to capture human-expert consensus across the four core metabolic engineering hierarchies—from global host selection to advanced dynamic control mechanisms—while effectively suppressing the potential for hallucination.

Beyond measuring the replication of established knowledge, we introduced the SynBioGPT Expansion Factor to characterize the model's capacity for autonomous innovation and its ability to explore the underexplored metabolic design space. This factor is defined as the ratio of the total number of theoretically feasible strategies generated by the AI system ($N_{\text{SynBioGPT}2}$) to the total number of strategies documented in the seminal literature ($N_{\text{literature}}$): Expansion Factor = $N_{\text{SynBioGPT}2}/N_{\text{literature}}$. This multiplier provides a quantitative measure of the system's generative breadth, distinguishing it from conventional retrieval-based engines by highlighting its utility in proposing combinatorial strategies, such as dynamic feedback loops and synthetic sRNA regulation, which may offer novel solutions to complex metabolic bottlenecks for subsequent wet-lab validation.

## 3 Results

### 3.1 Architectural ablation demonstrates the necessity of dynamic iteration for complex metabolic reasoning

**Fig. 1:**
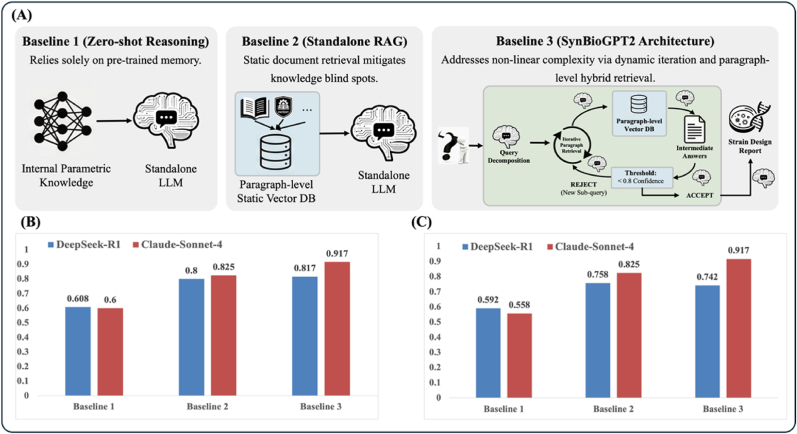
Architectural ablation and multidimensional validation of SynBioGPT2. (A) Schematic representation of the progressive architectural baselines for biological knowledge retrieval and synthesis. Baseline 1 represents the zero-shot reasoning limits of standalone LLMs relying solely on pre-trained parametric knowledge. Baseline 2 illustrates static paragraph-level hybrid retrieval for mitigating knowledge gaps. Baseline 3 depicts the complete SynBioGPT2 architecture, which employs query decomposition, paragraph-level hybrid retrieval, and a dynamic iteration loop with self-critique to resolve non-linear metabolic networks. (B, C) Performance comparison of DeepSeek-R1 and Claude-Sonnet-4 across the three baselines on a 120-item synthetic biology benchmark, quantified by accuracy (B) and completeness (C) (n = 120, Supplementary File 3).

To systematically evaluate the performance of the proposed architecture in resolving complex biological queries, we conducted an ablation study using a curated 120-item multidimensional synthetic biology benchmark (Supplementary File 4). Two state-of-the-art LLMs, DeepSeek-R1 [23] and Claude-Sonnet-4 (Claude-Sonnet-4-20250514), were assessed across three progressively complex architectural baselines (Fig. 1).

In the absence of external knowledge augmentation (Baseline 1), both models demonstrated baseline zero-shot reasoning capacities strictly limited by their pre-trained parametric knowledge (Fig. 1A). Under this setting, both models achieved an average accuracy of approximately 60.0% (DeepSeek-R1: 60.83%; Claude-Sonnet-4: 60.00%) (Fig. 1B). Notably, Claude-Sonnet-4 exhibited a reduced completeness score of 55.83% (Supplementary File 4; Fig. 1C). This conservative output generation is likely a byproduct of strict safety alignment mechanisms, wherein the model preferentially yields partial answers to mitigate hallucinations in low-confidence biological contexts.

To address the intrinsic knowledge deficits of standalone models, we integrated a robust static RAG pipeline featuring a two-stage hybrid retrieval system (Baseline 2) (Fig. 1A). This conventional retrieval-augmented approach substantially improved absolute accuracy by ∼20% (DeepSeek-R1: 80.00%; Claude-Sonnet-4: 82.50%), confirming the essential role of external domain-specific corpora (Supplementary File 4; Fig. 1B). However, detailed performance analysis revealed that even with advanced hybrid indexing, this static RAG architecture struggled significantly with multi-hop biochemical reasoning tasks (Supplementary File 4). Single-pass retrieval paradigms proved inadequate for capturing the causal dependencies required to computationally assemble fragmented metabolic pathways spanning multiple independent literature sources. Because biological systems are highly interconnected, an initial user query often cannot preemptively capture downstream regulatory feedback, rendering static retrieval susceptible to pathway-context misalignment.

Finally, we evaluated the complete SynBioGPT2 architecture (Baseline 3). Moving beyond static information retrieval, this configuration relies on dynamic query decomposition and an iterative self-evaluation loop (Fig. 1A). By leveraging Claude-Sonnet-4's extended context window and robust instruction-following capabilities, this configuration achieved the highest performance, elevating both accuracy and completeness to 91.67% (Supplementary File 4; Fig. 1B and C). These findings demonstrate that overcoming the non-linear complexity of metabolic regulatory networks cannot be reliably achieved through single-pass retrieval; rather, it necessitates the dynamic self-critique and iterative refinement mechanisms embedded within the SynBioGPT2 framework.

### 3.2 Task-specific benchmarking reveals the necessity of structured context for complex biochemical reasoning

**Fig. 2:**
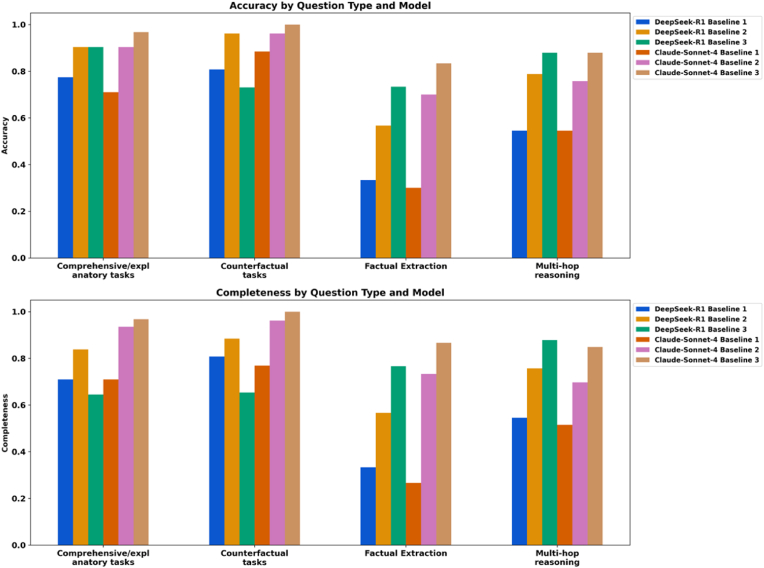
Performance breakdown across multidimensional synthetic biology tasks. Average accuracy (top) and completeness (bottom) scores are presented for four distinct biological reasoning categories: Comprehensive/explanatory, Counterfactual, Factual extraction, and Multi-hop reasoning. The performance of DeepSeek-R1 and Claude-Sonnet-4 is compared across three progressively complex architectural baselines (Baseline 1: standalone LLM; Baseline 2: Static RAG; Baseline 3: SynBioGPT2).

Building upon the overall architectural validation, we systematically evaluated performance across distinct types of biological queries. The benchmark dataset was categorized into four representative task dimensions, and output accuracy and completeness were assessed across all baselines (Supplementary File 4; Fig. 2). The results indicate that different biological tasks pose varying analytical challenges, with the complete SynBioGPT2 architecture (Baseline 3) demonstrating a critical advantage in processing highly specialized synthetic biology logic and bridging specific knowledge gaps.

For factual extraction tasks, models lacking external knowledge augmentation exhibited substantial deficits. Under Baseline 1, the accuracies of DeepSeek-R1 and Claude-Sonnet-4 were limited to 33.3% and 30.0%, respectively (Fig. 2). This poor performance highlights the difficulty of retrieving highly specialized, long-tail entities (e.g., specific plasmids, locus-specific point mutations) relying solely on internal parametric knowledge. While standard retrieval-augmented generation (Baseline 2) improved accuracies to 56.7%–70.0%, the SynBioGPT2 architecture (Baseline 3) yielded the most substantial improvements, raising the accuracies of DeepSeek-R1 and Claude-Sonnet-4 to 73.3% and 83.3%, respectively (Fig. 2). These results underscore the necessity of deep indexing and structured tool orchestration for the high-fidelity extraction of specialized biological facts.

Multi-hop reasoning tasks require establishing causal connections across disjointed biological concepts or pathways. In this category, Baseline 1 plateaued at an accuracy of ∼54.5%, and static hybrid RAG (Baseline 2) frequently failed to connect necessary logical steps, as it lacks the capacity to dynamically generate subsequent sub-queries based on retrieved intermediate evidence (Fig. 2). In contrast, the SynBioGPT2 architecture increased the accuracy of both models to 87.9% (Fig. 2). By providing a structured, relational context, this framework facilitates the reconstruction of fragmented logical chains, enabling the models to accurately infer indirect causal relationships within complex metabolic or regulatory networks.

In comprehensive and explanatory tasks, advanced LLMs demonstrated a strong baseline capacity for semantic integration (Baseline 1 accuracies: 71.0%–77.4%) (Fig. 2). The integration of SynBioGPT2 with Claude-Sonnet-4 further elevated accuracy to 96.8% (Fig. 2). However, DeepSeek-R1 exhibited a slight reduction in completeness (64.5%) under Baseline 3 compared to Baseline 2 (Fig. 2). This suggests that when presented with high-density, multi-source retrieved literature, certain models may struggle to perfectly synthesize all provided context, occasionally resulting in the omission of specific explanatory dimensions.

Counterfactual tasks, designed to evaluate logical discrimination and hypothesis testing, revealed distinct model-specific behaviors. While Baseline 2 generally improved accuracies to 96.2%, the SynBioGPT2 architecture enabled Claude-Sonnet-4 to correctly resolve all counterfactual queries (100% accuracy) (Fig. 2). Conversely, DeepSeek-R1's accuracy decreased to 73.1% under the same framework (Fig. 2). This performance divergence indicates that extensive external factual inputs can occasionally conflict with a model's internal pre-trained logic during counterfactual reasoning. Balancing the integration of external evidence with internal hypothesis evaluation remains a critical consideration for optimizing these computational frameworks across diverse biological scenarios.

### 3.3 Resolving multi-scale metabolic engineering challenges through dynamic context retrieval

To validate the practical utility of the SynBioGPT2 architecture beyond statistical benchmarks, we evaluated its performance in rational strain design and knowledge extraction using *Corynebacterium glutamicum* as a model industrial chassis (Table 1).

**Table 1:** Performance comparison of SynBioGPT2 against conventional frameworks across multi-scale metabolic engineering scenarios.

Engineering Scale	Case Study	Core Biological/Analytical Challenge	Conventional LLM Limitations	SynBioGPT2 Resolution Strategy
Systems Biology (Macro-scale)	L-valine overproduction in *C. glutamicum*	Cofactor imbalances and systemic regulatory feedback	Fails to account for non-linear metabolic responses and dynamic global feedback	Employs dynamic iteration to map complete causal chains
Protein Engineering (Micro-scale)	Redirecting carbon flux toward L-isoleucine	Severe allosteric feedback inhibition of threonine dehydratase	Consistently overlooks micro-level structural modifications and synergistic mechanisms	Utilizes targeted sparse indexing to identify critical single-amino-acid substitutions alongside genomic edits
Quantitative Data (Data-scale)	Titer extraction for L-carnosine biosynthesis	High-fidelity numerical traceability amidst complex intermediate and final strain construction data	Confounding of basal production, intermediate titers, and final yield parameters.	Enforces paragraph-level indexing and strict search boundaries to extract precise, strain-specific metrics.

At the systems biology level, optimizing *C. glutamicum* for L-valine overproduction presents significant challenges, primarily regarding cofactor imbalances and systemic regulatory feedback (Supplementary File 4, Question 40). Traditional static retrieval systems frequently fail to account for non-linear metabolic responses, such as secondary allosteric inhibition triggered by the deletion of competing pathways (e.g., the *ppc* gene). In contrast, by employing dynamic iteration, the SynBioGPT2 architecture successfully formulated a comprehensive, mechanistically sound metabolic strategy. It correctly linked cofactor remodeling and byproduct pathway deletion with the necessary targeted rescue of glycolytic flux to alleviate global allosteric inhibition, demonstrating its capacity to navigate systems-level metabolic network dependencies.

The architecture's precision was further validated at the protein engineering level during the redirection of carbon flux towards L-isoleucine biosynthesis (Supplementary File 4, Question 93). A major bottleneck in this pathway is the severe allosteric feedback inhibition of threonine dehydratase (*ilvA*). While conventional LLM architectures consistently overlooked specific structural modifications, the targeted retrieval module of SynBioGPT2 accurately identified the critical single-amino-acid substitution (F383V) and contextualized its synergistic application with the *ddh* and *lysE* locus deletions [24,25]. This capability to integrate macro-level genomic edits with micro-level point mutations indicates that the system can effectively resolve complex genotype-phenotype relationships.

Finally, we assessed the framework's reliability in quantitative data extraction, a task historically plagued by factual inconsistencies or “hallucinations” in standalone LLMs. When querying the L-carnosine titer achieved via *panD* overexpression (Supplementary File 4, Question 2), baseline models frequently confounded basal production, intermediate titers, and final yields. By enforcing paragraph-level indexing and strict search boundaries, SynBioGPT2 bypassed contextual noise to accurately extract the specific titer (99.17 mg/L) associated with the intermediate strain Car13 [26]. This high-fidelity extraction of quantitative parameters is crucial for establishing accurate boundary conditions for computational strain design and industrial scale-up models.

### 3.4 Validation of SynBioGPT2 in rational strategy generation and metabolic design space expansion

**Fig. 3:**
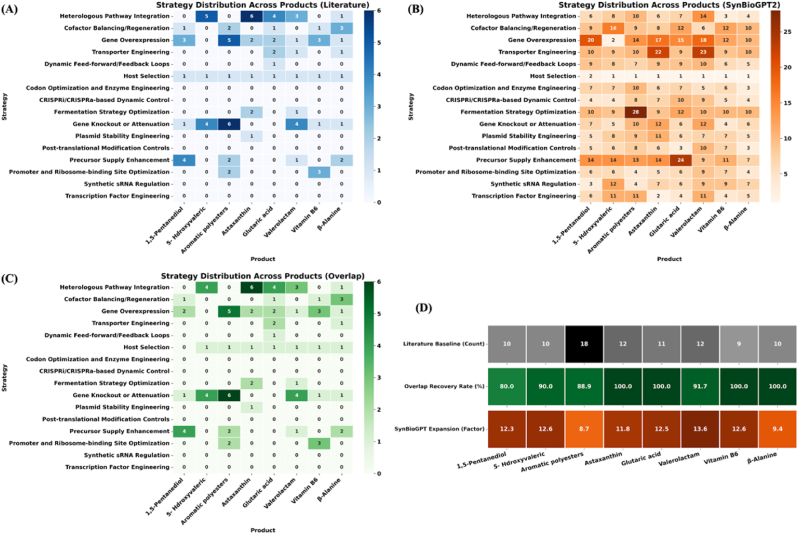
Multidimensional evaluation of metabolic engineering strategies and design space expansion by SynBioGPT2. (A-C) Heatmaps of strategy distribution. The matrices display the frequency of specific metabolic interventions across eight target products derived from (A) seminal literature baselines (blue), (B) SynBioGPT2 autonomous generation (orange), and (C) their intersection (Overlap, green). Color intensities denote the recommendation weight of specific strategies. (D) Multi-metric performance evaluation matrix assessing the model's fidelity and generative capacity. The top panel (grey) indicates the absolute count of expert-curated literature strategies. The middle panel (green) displays the Overlap Recovery Rate (%), representing the successful reconstruction of historical consensus. The bottom panel (orange) illustrates the Design Space Expansion Factor, quantifying the ratio of AI-generated theoretical strategies to the literature baseline to evaluate the system's capacity for identifying novel metabolic interventions.

To comprehensively evaluate the capacity of SynBioGPT2 to function as an autonomous “AI Scientist” for long-horizon metabolic engineering tasks, we benchmarked its generative output against a curated dataset of 92 high-value metabolic engineering strategies. These strategies, extracted from seminal literature across eight representative target products (ranging from bulk chemicals like 1,5-pentanediol to high-value compounds like astaxanthin), reflect established human-expert consensus (Fig. 3A) [[27], [28], [29], [30], [31], [32], [33], [34]]. Guided by 16-element expert prompt templates (Supplementary File 2), SynBioGPT2 successfully reconstructed 86 of the 92 strategies, achieving an overall recovery rate of 93.5% (Fig. 3C and D). The substantial overlap in the strategy distribution heatmaps (Fig. 3D, green zones) demonstrates the framework's ability to robustly capture historical biological prior knowledge. In classical design dimensions heavily reliant on expert intuition—such as heterologous pathway integration and gene overexpression—the model effectively anchored its design logic in validated biological principles rather than stochastic text generation (Supplementary Files 5 and 6).

In-depth analysis of the successfully reconstructed strategies confirmed that SynBioGPT2's outputs seamlessly align with classical rational design frameworks across four core metabolic hierarchies (Fig. 3C; Supplementary File 5). At the global systems level, the framework demonstrated macro-level logic by optimizing host selection and fermentation strategies. For pathway reconstruction, it exhibited precise flux control, actively recommending heterologous pathway integration and competitive pathway attenuation to alleviate intermediate toxicity. At the regulatory tier, the system accurately prescribed strategies for cofactor balancing and the alleviation of allosteric feedback inhibition via promoter and ribosome-binding site optimization. Finally, at the advanced dynamic control level, the framework successfully deployed complex mechanisms, including transporter engineering for product efflux and plasmid stability maintenance.

Crucially, the utility of SynBioGPT2 extends significantly beyond the passive retrieval of historical knowledge by actively expanding the metabolic engineering design space (Fig. 3D, bottom panel). The strategy distribution matrix (Fig. 3B, orange zones) reveals that the framework autonomously generated a substantially broader array of theoretically viable strategies compared to the literature baseline (Fig. 3A, blue zones). While traditional experimental designs are often constrained by the prohibitive costs of trial-and-error—typically resulting in local optimizations of a few rate-limiting enzymes—SynBioGPT2 formulates global, systems-level solutions. For example, in the biosynthesis of complex molecules like aromatic polyesters and valerolactam, the system not only reconstructed standard pathway modifications but also introduced dense regulatory networks for cofactor regeneration and product efflux (Fig. 3B). Furthermore, transitioning away from a heavy reliance on static gene knockouts, SynBioGPT2 systematically integrated cutting-edge combinatorial strategies—such as CRISPRi/a-based dynamic control and synthetic sRNA regulation—providing highly innovative targets for overcoming complex multi-gene regulatory bottlenecks (Fig. 3B).

Collectively, this high-resolution evaluation validates the evolution of SynBioGPT2 from a conventional retrieval tool into an “AI Scientist” capable of executing complex, long-horizon biological designs. By autonomously discovering non-intuitive metabolic engineering strategies, this framework facilitates a fundamental paradigm shift in synthetic biology: transitioning from the traditional, human-experience-dependent DBTL cycle to an “AI-guided Design (LD) - Human/Automated Test (BT)" paradigm. This data-driven digital engine establishes a robust computational foundation for the rapid, systems-level development of future customized cell factories.

## 4 Discussion

The rational design of microbial cell factories is challenged by the complex, non-linear nature of biological systems, where localized genetic perturbations often induce systemic shifts in metabolic fluxes and regulatory networks. While LLMs offer significant natural language processing capabilities, their direct application to systems metabolic engineering is constrained by factual inaccuracies and a limited capacity for multi-hop reasoning. The SynBioGPT2 architecture addresses these limitations by shifting from static information retrieval to a dynamic, iterative reasoning framework, providing a robust computational approach for comprehensive strain engineering.

Our ablation study highlights the parametric constraints of standalone LLMs in scientific reasoning. Models such as Claude-Sonnet-4 and DeepSeek-R1 exhibited limited efficacy in complex, multi-hop biological tasks without external knowledge augmentation (Baseline 1). Furthermore, we demonstrated that even a robust, two-stage hybrid RAG pipeline (Baseline 2) proved insufficient for assembling causal chains across disparate literature sources. Because biochemical mechanisms are rarely confined to single text segments, single-pass retrieval cannot proactively fetch necessary downstream regulatory context. The superior performance of the complete SynBioGPT2 architecture (Baseline 3) indicates that integrating paragraph-level hybrid retrieval with a dynamic iteration loop is essential for navigating these complexities. By iteratively evaluating intermediate outputs and retrieving missing contextual data, SynBioGPT2 computationally mimics the deductive reasoning processes required for multi-source knowledge synthesis.

This capability for structural context integration was demonstrated in the rational design of *C*. *glutamicum*. Optimizing the biosynthesis of highly regulated metabolites, such as L-valine and L-isoleucine, requires precise navigation of thermodynamic and regulatory constraints. Traditional static retrieval systems frequently fail to capture systems-level dependencies, such as interpreting a *ppc* deletion solely as a yield-enhancing strategy while omitting the subsequent NADH accumulation and allosteric inhibition of GAPDH. Through dynamic context retrieval, SynBioGPT2 successfully resolved these biochemical dependencies, proposing necessary compensatory modifications such as *gapA* overexpression. Similarly, the system's accurate identification of the F383V point mutation in *ilvA* to bypass feedback inhibition highlights its proficiency in addressing both macro-level flux redirection and micro-level protein engineering targets.

The transition from laboratory-scale engineering to industrial biomanufacturing relies heavily on precise quantitative parameters for techno-economic analysis and scale-up modeling. Generalized LLMs frequently exhibit feature conflation during quantitative extraction, leading to inaccurate parameter retrieval across different operational scales or strains. SynBioGPT2 mitigates these inconsistencies through strict paragraph-level indexing and defined search boundaries. As shown in the L-carnosine biosynthesis case study, the architecture accurately extracted the intermediate titer of 99.17 mg/L specific to *panD* overexpression, effectively isolating it from contextual noise such as final fed-batch yields. This high-fidelity data extraction provides the rigorous reliability necessary for automated computational strain design.

Beyond algorithmic accuracy, the practical adoption of an automated design framework by the broader metabolic engineering community depends critically on its computational accessibility and operational cost. Using the DeepSeek-R1 API, an 8-cycle task (e.g., L-carnosine design) completes in ∼2 min, utilizing 114,086 tokens for a total cost of $0.06 USD. Local hosting of the vector database and BM25 index ensures near-zero retrieval latency. Ultimately, SynBioGPT2 offers routine academic and industrial accessibility with operational costs comparable to basic molecular biology reagents.

Having established both the analytical precision and the computational feasibility of the framework, we next evaluated its capacity for autonomous strategy generation and metabolic design space expansion. The framework's utility for systems-level design is further supported by its successful reconstruction of 86 out of 92 (93.5%) expert-curated metabolic engineering strategies. The model demonstrated computational proficiency across core metabolic hierarchies, ranging from global host selection to advanced regulatory control and transporter engineering. Guided by structured expert prompt templates, the generated strategies aligned tightly with established mechanistic principles rather than stochastic text associations. By effectively anchoring its logic in validated biological principles, the framework not only standardizes the computational design process but also expands the theoretical design space for identifying novel metabolic engineering strategies.

While SynBioGPT2 substantially broadens the theoretical design space, this study currently lacks in vivo experimental validation for the autonomously generated combinatorial strategies. Nevertheless, within the scope of rational computational design, the framework serves as a robust hypothesis-generation engine. Conventional DBTL cycles are frequently constrained by the combinatorial explosion of the perturbation space. By grounding its generative outputs in established biochemical mechanisms, SynBioGPT2 systematically eliminates biologically inviable configurations. This targeted reduction of the search space yields a refined set of high-confidence, actionable interventions, thereby accelerating the upstream 'Design' phase and optimizing resource allocation for downstream empirical testing.

Beyond this empirical constraint, several computational limitations also remain. First, SynBioGPT2 is inherently bounded by the existing corpus of published literature; it cannot autonomously predict undocumented enzymatic functions or novel structural dynamics without integration with physics-based predictive models (e.g., AlphaFold3 [35] or ESM-3 [20]) or computational chemistry frameworks [[36], [37], [38]]. Second, while the system qualitatively resolves metabolic flux imbalances, it currently lacks the capability to execute quantitative steady-state simulations. Future developments will focus on interfacing the SynBioGPT2 inference module directly with advanced multi-constraint genome-scale metabolic models (GEMs). By integrating algorithms such as enzyme-constrained modeling (e.g., ECMpy [39,40]) or multidimensional constraint frameworks (e.g., ETGEMs [41]), the pipeline could enable real-time, quantitative flux balance analysis (FBA) to dynamically validate the specific genetic perturbations proposed by the AI prior to finalizing the strategy blueprint.

In conclusion, SynBioGPT2 presents a systematic approach to integrating LLMs into synthetic biology by mitigating structural reasoning deficits and factual inaccuracies. This architecture provides a scalable, mechanistically grounded platform for automated biological design. As the biomanufacturing sector increasingly adopts automated workflows and “Cloud Labs”, dynamic reasoning frameworks like SynBioGPT2 will be critical for generating the high-fidelity, systems-level blueprints necessary to advance rational cell factory engineering.

## 5 Conclusion

In this study, we developed the SynBioGPT2 architecture to address the structural reasoning deficits and factual inconsistencies that limit the application of generalized LLMs in systems metabolic engineering. By integrating a 16-element expert prompt template, paragraph-level hybrid retrieval, and a dynamic iteration loop, the framework successfully navigates the complex, non-linear dependencies of microbial cell factory design. SynBioGPT2 effectively resolved numerical conflation during quantitative data extraction and achieved a 93.5% success rate in reconstructing expert-curated, systems-level metabolic interventions. These performance metrics demonstrate a critical advancement from static single-pass retrieval to mechanistically rigorous computational design.

This capability has significant implications for the traditional DBTL cycle. As synthetic biology increasingly scales toward high-throughput, automated “Cloud Labs”, the primary bottleneck has shifted from physical experimental execution to rational, systems-level design. By standardizing the human-AI interaction interface and ensuring the high-fidelity extraction of parameters necessary for techno-economic and scale-up models, SynBioGPT2 establishes the reliable boundary conditions required for capital-efficient industrial biomanufacturing. Ultimately, this human-AI collaborative framework bridges the gap between fragmented biological literature and holistic cell factory optimization, providing a scalable and robust computational platform for the next generation of automated biological engineering.

## Author contributions

Z.M., X.L. and H.M. designed the study and wrote the manuscript. J.D, R.W. designed the architecture and deployment of SynBioGPT2. H.L. developed the frontend interface of SynBioGPT2. Z.M., J.G., Z.S., W.W. and Q.Y. collected synthetic biology-related data and conducted testing on SynBioGPT2. All authors have read and approved the final manuscript.

## Data availability

The SynBioGPT2 web platform is publicly accessible at https://synbiogpt.biodesign.ac.cn/. The 120-item multidimensional synthetic biology benchmark dataset generated and analyzed during the current study is available in Supplementary File 3. For readers less familiar with artificial intelligence and computational terminology, a comprehensive glossary mapping each technical term to microbiology-specific analogies is provided in Supplementary File 7. The domain-specific corpus utilized for paragraph-level indexing was compiled from open-access literature repositories, including Web of Science and PubMed Central.

## Code availability

The expert prompt templates for strain design (Supplementary File 2) and the evaluation prompts for the LLM-as-a-Judge pipeline (Supplementary File 3) are provided with this paper. The core inference scripts, and automated evaluation pipelines utilized for the SynBioGPT2 architecture are available from the corresponding author upon reasonable request.

## Declaration of competing interest

The authors declare the following financial interests/personal relationships which may be considered as potential competing interests:Given his role as Associate Editor, Hongwu Ma had no involvement in the peer review of this article and had no access to information regarding its peer review. Full responsibility for the editorial process for this article was delegated to another journal editor.
