# Supplementary material for: SynBioGPT2: A dynamic reasoning framework enables high-fidelity design of microbial cell factories
Source: Biodes Res. 2026 Jun 25;8(3):100093. doi: 10.1016/j.bidere.2026.100093 (PMC13377148; doi:10.1016/j.bidere.2026.100093)
Supplement: Multimedia component 1 [file mmc1.docx]

Table S1 Comparative analysis of SynBioGPT1 and SynBioGPT2

| Dimension | SynBioGPT1 | SynBioGPT2 |
| --- | --- | --- |
| Knowledge Base | Static corpus, limited updates | Regularly updated corpus |
| Preprocessing | Basic document storage | Structured, metadata-rich Markdown with OCR, table parsing, and semantic segmentation |
| Indexing Strategy | Semantic embeddings | Hybrid retrieval combining sparse BM25 and dense BERT embeddings |
| Granularity of Indexing | Document-level | Paragraph-level |
| Query Understanding | Direct embedding-based retrieval | LLM-based reasoning: interprets intent, formulates sub-questions |
| Query Decomposition | None | Iterative, evidence-driven decomposition and refinement |
| Answer Evaluation | No self-assessment | Self-evaluation of answer adequacy against original query |
| Reasoning Workflow | None | Iterative reasoning |
| Risk of Hallucination | Higher, due to broad semantic matches | Reduced, via iterative reasoning and confidence thresholds |
| Precision and Coverage | Broad coverage, lower precision | Balanced precision and recall, higher contextual relevance |
