# Supplementary material for: SynBioGPT2: A dynamic reasoning framework enables high-fidelity design of microbial cell factories
Source: Biodes Res. 2026 Jun 25;8(3):100093. doi: 10.1016/j.bidere.2026.100093 (PMC13377148; doi:10.1016/j.bidere.2026.100093)
Supplement: Multimedia component 2 [file mmc2.docx]

**Prompt templates of metabolic engineering strategies**

**Host Selection**

**1.Host Selection**

Concurrently optimizing genetic tractability, metabolic network compatibility, and industrial scalability, identify the optimal host organism (bacterial/yeast/algal) for industrial scale glutaric acid production.

**2.Heterologous pathway integration**

In the metabolic engineering of *Corynebacterium glutamicum* for glutaric acid production, which specific heterologous enzymes or biosynthetic pathways can be integrated to complement host metabolism and enhance the overall biosynthetic efficiency?

**Metabolic Pathway Engineering**

**1.Cofactor Balancing/Regeneration**

How can the optimization of cofactor balancing and regeneration pathways in *Corynebacterium glutamicum* enhance the production of glutaric acid, and what specific strategies or genetic modifications can be employed to maintain optimal cofactor levels for efficient biosynthesis?

**2.Gene Overexpression**

Which specific genes involved in the glutaric acid biosynthetic pathway or related metabolic processes should be overexpressed in *Corynebacterium glutamicum* to maximize the efficiency, yield, and overall output of glutaric acid production?

**3.Gene Knockout or Attenuation**

In the metabolic engineering of *Corynebacterium glutamicum* for glutaric acid production, which specific genes can be knocked out or attenuated to optimize the production efficiency of glutaric acid by redirecting metabolic flux and minimizing the formation of undesirable byproducts?

**4.Codon optimization and enzyme engineering**

What codon optimization strategies and enzyme engineering approaches—particularly through synonymous codon substitution and targeted point mutations—can be applied in *Corynebacterium glutamicum* to improve the expression level, folding efficiency, and catalytic performance of key enzymes in the glutaric acid biosynthetic pathway?

**5.Precursor supply enhancement**

In *Corynebacterium glutamicum*, how can precursor supply enhancement strategies be applied to increase the intracellular availability of key metabolic precursors for glutaric acid production?

**6.Transporter Engineering**

In *Corynebacterium glutamicum*, how can transporter engineering be applied to enhance the transmembrane transport of key substrates, products, and inhibitors in glutaric acid production, and which specific transporters and regulatory mechanisms should be targeted to improve substrate uptake, facilitate product export, and mitigate issues such as byproduct accumulation, product toxicity, and nutrient limitations?

**Metabolic Pathway Regulation**

**1.Dynamic feed‐forward/feedback loops**

In *Corynebacterium glutamicum*, how can dynamic feed-forward and feedback regulation be engineered to relieve feedback inhibition and optimize the availability of metabolic substrates in the glutaric acid biosynthetic pathway?

**2.Transcription factor engineering**

Which transcription factors should be modified or overexpressed in *Corynebacterium glutamicum* to redirect the transcriptional network toward the glutaric acid biosynthetic pathway, enhancing the expression of key pathway genes while reducing flux to competing metabolic routes?

**3.Promoter and ribosome-binding site (RBS) optimization**

In the metabolic engineering of *Corynebacterium glutamicum* for glutaric acid production, how can promoter and ribosome-binding site (RBS) optimization be strategically applied to balance enzyme expression, alleviate metabolic bottlenecks, and improve overall pathway efficiency and product yield?

**4.CRISPRi/CRISPRa‐based dynamic control**

In the metabolic engineering of *Corynebacterium glutamicum* for glutaric acid production, how can CRISPRi/CRISPRa-based dynamic control systems be leveraged to precisely regulate gene expression timing and levels, enabling the redistribution of cellular resources toward glutaric acid synthesis during optimal growth phases while minimizing by-product formation?

**5.Synthetic sRNA regulation**

In the metabolic engineering of *Corynebacterium glutamicum* for glutaric acid production, how can synthetic small RNAs (sRNAs) be designed to achieve precise post-transcriptional regulation, enabling the downregulation of competing pathways and fine-tuning of key enzyme expression to redirect carbon flux and enhance glutaric acid biosynthesis?

**6.Post‐translational modification (PTM) controls**

In the metabolic engineering of *Corynebacterium glutamicum* for glutaric acid production, how can post-translational modifications (PTMs) such as phosphorylation or acetylation be leveraged to dynamically regulate the activity and stability of key biosynthetic enzymes, and which targets and strategies are most effective for modulating metabolic flux in response to cellular signals or environmental changes to optimize product yield?

**Experimental Condition Optimization**

**1.Plasmid Stability Engineering**

How can plasmid stability be engineered in *Corynebacterium glutamicum* to ensure reliable retention of glutaric acid production plasmids during cell division, and what strategies can minimize plasmid loss across generations to maintain consistent biosynthetic output?

**2.Fermentation Strategy Optimization**

What fermentation strategy optimization techniques can be applied to improve key performance metrics, such as yield, productivity, and growth efficiency in *Corynebacterium glutamicum* for glutaric acid production, while minimizing factors like toxicity, cost, or inhibitory effects on the system?
