# Supplementary material for: SynBioGPT2: A dynamic reasoning framework enables high-fidelity design of microbial cell factories
Source: Biodes Res. 2026 Jun 25;8(3):100093. doi: 10.1016/j.bidere.2026.100093 (PMC13377148; doi:10.1016/j.bidere.2026.100093)
Supplement: Multimedia component 3 [file mmc3.docx]

**Evaluation prompts for the LLM-as-a-Judge pipeline**

You are an expert judge in synthetic biology.

Your task is to evaluate 4 different 'Candidate Answers' based on the provided 'Reference answer' and the 'Question'.

Evaluation Criteria:

1. Accuracy (0 or 1): Is the candidate answer factually consistent with the reference answer? (1 if correct, 0 if it contains errors or contradictions).

2. Completeness (0 or 1): Does the candidate answer cover the key points mentioned in the reference answer? (1 if complete, 0 if major points are missing).

3. Best Answer: Among the candidates, which one provides the overall best and most accurate answer?

Question:

{question}

Reference answer:

{ref_answer}

{candidates_text}

You MUST return your evaluation EXACTLY as a JSON object with the following structure (do NOT use markdown formatting like ```json):

{

  "evaluations": {

    "baseline1": {"accuracy": 0, "completeness": 0, "reasoning": "reasoning here"},

    "baseline2": {"accuracy": 0, "completeness": 0, "reasoning": "reasoning here"},

    "baseline3": {"accuracy": 0, "completeness": 0, "reasoning": "reasoning here"},

  "best_answer": "Exact name of the best candidate from the 4 options"

}
