# Supplementary material for: SynBioGPT2: A dynamic reasoning framework enables high-fidelity design of microbial cell factories
Source: Biodes Res. 2026 Jun 25;8(3):100093. doi: 10.1016/j.bidere.2026.100093 (PMC13377148; doi:10.1016/j.bidere.2026.100093)
Supplement: Multimedia component 7 [file mmc7.docx]

**Glossary of Technical Terms for Biological Readership**

To enhance accessibility for the broad biological readership of BioDesign Research, we provide below a glossary of specialized computational and AI terminology used in the manuscript. Each term is explained using microbiology-specific analogies and established concepts from the field.

**1. Multi-hop reasoning**

A reasoning process that requires connecting information across multiple intermediate steps to reach a final conclusion. In microbiology, this is analogous to tracing a complete metabolic pathway across multiple enzymatic reactions (e.g., glucose → pyruvate → acetyl-CoA → fatty acids), where each step requires integrating knowledge from a different gene, enzyme, or regulatory node. Just as understanding fatty acid overproduction requires knowledge of upstream glycolysis, multi-hop reasoning links disjointed literature evidence into a coherent biological strategy.

**2. Retrieval-augmented generation (RAG)**

A framework that grounds large language model outputs in external, verifiable knowledge sources rather than relying solely on internal parametric memory. This is conceptually similar to a researcher consulting multiple databases (PubMed, KEGG, BRENDA, or RegPrecise) during experimental design to ensure that a proposed metabolic engineering strategy is supported by published evidence, rather than relying only on personal memory or intuition.

**3. Multidimensional synthetic biology benchmark**

A standardized evaluation suite that assesses an AI system across multiple distinct task categories. Analogous to a multi-stress tolerance assay (e.g., simultaneously testing thermotolerance, osmotolerance, and acid tolerance in a single strain panel), this benchmark evaluates performance across factual extraction, multi-hop reasoning, comprehensive explanation, and counterfactual rejection.

**4. Multidimensional perturbation space**

The combinatorial landscape of all possible genetic and environmental modifications applicable to a microbial chassis. This includes gene knockouts, overexpression, promoter/RBS optimization, CRISPRi/a dynamic regulation, and media composition changes. It is conceptually similar to the design space explored in media optimization or DOE (Design of Experiments), where multiple factors (carbon source, nitrogen source, pH, temperature) are varied simultaneously to identify optimal production conditions.

**5. Single-pass paradigm**

An information retrieval approach in which the system performs one round of document search and immediately generates an answer without subsequent refinement. This is analogous to performing a single PCR cycle without optimization or performing a one-shot fermentation without iterative process development. In contrast, iterative methods (like the DBTL cycle) allow progressive refinement based on intermediate outcomes.

**6. Paragraph-level hybrid indexing**

A search strategy that indexes scientific literature at the paragraph (rather than document) level, treating each paragraph as an independent knowledge unit. This is analogous to analyzing individual wells in a 96-well plate rather than averaging an entire plate, enabling precise localization of specific experimental observations. The 'hybrid' aspect combines keyword matching (BM25, similar to a PubMed title/abstract search) with semantic embedding (similar to sequence homology via BLAST), maximizing both precision and recall.

**7. Iterative self-evaluation loop**

A dynamic reasoning mechanism in which the AI system critiques its own intermediate outputs, identifies knowledge gaps, and autonomously retrieves additional evidence to refine its conclusions. This mirrors the iterative nature of the Design-Build-Test-Learn (DBTL) cycle, where each experimental round informs the next round of hypothesis refinement and genetic modification.

**8. High-fidelity design**

In the context of this manuscript, 'high-fidelity' refers to the precision, accuracy, and reliability with which the computational framework translates fragmented biological knowledge into mechanistically rigorous, actionable engineering strategies. It does not imply perfect replication of a pre-existing biological template (as in high-fidelity PCR), but rather denotes faithful representation of biochemical constraints and quantitative accuracy in de novo engineering designs, analogous to how high-fidelity computational simulations faithfully model physical systems.

**9. Dynamic reasoning**

A computational approach that adapts its inference strategy in real-time based on intermediate results, rather than following a fixed, predetermined sequence. In metabolic engineering, this is analogous to adaptive laboratory evolution (ALE) or dynamic metabolic control, where the system responds to real-time metabolite concentrations or physiological states to optimize production, rather than applying static, constitutive genetic modifications.

**10. LLM-as-a-Judge**

An evaluation methodology in which an independent large language model (distinct from the system being evaluated) assesses the factual accuracy and completeness of generated outputs. This is analogous to blinded peer review or independent validation experiments, where an external expert (or a different laboratory) evaluates the reproducibility and validity of published results to mitigate confirmation bias.

**11. Query decomposition**

The process of breaking a complex biological design question into smaller, sequentially dependent sub-questions. This mirrors the hierarchical modularity of metabolic engineering projects: a grand challenge (e.g., 'engineer a cell factory for astaxanthin') is decomposed into tractable sub-tasks (host selection, heterologous pathway integration, precursor supply optimization, cofactor balancing, and dynamic regulation), each addressed independently before final integration.

**12. Expert prompt template**

A structured, pre-defined query framework that guides users to interact with the AI system in a manner consistent with established metabolic engineering logic. Analogous to standardized laboratory protocols (SOPs) or experimental design templates (e.g., plasmid construction checklists), these templates ensure reproducibility and completeness by systematically covering the four core dimensions of strain design: host selection, pathway engineering, metabolic regulation, and experimental condition optimization.
